# Editorial: Microbial communities and microbiomes in dairy products

**DOI:** 10.3389/fmicb.2023.1265035

**Published:** 2023-08-08

**Authors:** Celia C. G. Silva, Susana C. Ribeiro, Benedetta Bottari

**Affiliations:** ^1^School of Agrarian and Environmental Sciences, University of the Azores, Angra do Heroismo, Portugal; ^2^Institute of Agricultural and Environmental Research and Technology (IITAA), Angra do Heroísmo, Portugal; ^3^Department of Food and Drug Sciences, University of Parma, Parma, Italy

**Keywords:** milk, dairy industry chain, dairy products, milk fermentation ability, cheese microbiota, microbial biodiversity, high-throughput sequencing (HTS)

Microbial communities in milk can be very complex and include numerous bacterial, yeast and fungal species originating from various sources of contamination, such as the external udder surface, milking equipment, air, water, feed, grass and soil. Some of these microorganisms play an important role in the dairy industry, especially in fermented foods, while others can cause spoilage or compromise the safety of dairy products. In fermented foods such as cheese, microbial communities contribute to the transformation of milk through their growth and metabolic activities. These collective genomes within microbial communities are essential for the production of many dairy products that are recognized and appreciated by consumers worldwide.

The study of whole microbial communities in dairy products has made significant progress in recent years. This has been driven by the development of high-throughput sequencing technologies that allow researchers to study the composition and function of microbial communities on a much larger scale than was previously possible. Advances in metagenomics and metatranscriptomics research have revealed the functional potential of individual microorganisms as well as entire communities in their habitats. Furthermore, fermented dairy products have been shown to have a positive impact on consumer health due to their rich and diverse microbial populations. Therefore, it is particularly important to explore the diversity of microbial communities and microbiomes in milk and dairy products, as well as their interactions and potential health benefits.

The main objective of this Research Topic was to uncover the microbial communities and microbiomes present in dairy products in order to better understand their role in the production and quality of these foods. Four papers have been published under this theme, adding to our knowledge of the presence and diversity of microbial communities in milk and cheese.

The composition of the microbiome of cheese can vary depending on several factors, including the type of milk used, the production process and the ripening conditions. Although the microbiota of cheese is complex, it is dominated by lactic acid bacteria (LAB), which play an important role both as starters and in cheese ripening. LAB can be used as defined starter cultures added to pasteurized milk, or they can arise as the natural microbiota of milk, as is the case with many artisanal cheeses made from raw milk. Indigenous LAB strains derived from the milk microbiota are selected for their ability to grow under the highly selective conditions that prevail during cheese ripening. These non-starter LAB (NSLAB) play a key role in the development of texture, aroma and flavor of cheeses.

The review by Bettera, Levante et al. examines the scientific literature regarding the population of LAB in raw milk used for cheese making, estimated using culture-dependent and culture-independent methods. By using a Next Generation Sequencing (NGS) approach, the genus *Lactococcus* was found to be the dominant LAB, while sub-dominant taxa included *Streptococcus, Lacticaseibacillus, Lactobacillus, Leuconostoc*, and *Enterococcus*. Overall, the research results using culture-dependent methods agreed with the culture-independent methods, as were found higher plate counts of *Lactococcus* and *Streptococcus* in raw milk compared to *Lactobacillus* and *Lacticaseibacillus* counts.

In a second article, Bettera, Dreier et al. address the need for effective supplementary cultures that can be used by artisanal cheesemakers to ensure the quality, reproducibility and consistency of artisanal cheese. In this paper, the authors tested different protocols to produce an enriched raw milk whey culture (eRWC) by co-fermenting raw milk enriched by spontaneous fermentation for 21 days at 10°C with a natural whey culture (NWC). Although further research is needed to optimize culture production, this study has shown that these enrichment protocols can increase the concentration of autochthonous LAB in cheese and could provide a tool to produce natural adjunct cultures for artisanal cheese production.

Several artisanal cheeses are now protected by a “protected designation of origin” (PDO), which legally defines the region and the production technology of these cheeses. These PDO cheeses have unique characteristics in terms of their artisanal production methods and are often characterized by a richer and more distinctive taste. The work of Levante et al. describes the dynamics characterizing the microbial ecology of Mozzarella di Bufala Campana PDO and evaluates the impact of different cheesemaking technologies on the cheese microbiome using NGS methods. They studied two production facilities that used different cheese making processes with traditional and automated technologies. This work considered different steps of cheese production, including brine analysis, to evaluate the dynamics of the mozzarella microbiome. The results showed no significant differences in the microbiome between production technologies, while the microbiota of this cheese was dominated by the genera *Streptococcus* and *Lactobacillus*. It was concluded that the composition of the NWS is the most important factor determining the composition of the microbiome of the finished mozzarella cheese.

In addition to bacteria, cheese also contains yeasts and molds that contribute to the ripening process either directly through their metabolic activity or indirectly through the release of enzymes into the cheese matrix. The manuscript by Martin et al. reports on a change in cheesemakers' practice of preserving the indigenous fungi that grow on cheese surface during the ripening of Brazilian artisanal Canastra cheese. The authors not only describe the yeast and filamentous fungal communities of this artisanal cheese using NGS, but also report on the influence of season, region and farm on the cheese mycobiota. The results presented in this study showed greater fungal diversity in cheeses made during the rainy season and provided important new insights into the importance of the presence of fungi in this artisanal cheese.

In summary, the microbial communities and microbiomes in dairy products are complex and diverse and play an important role in the production, flavor, and quality of these foods, especially PDO cheese. Microbial succession during cheese ripening is related to the ability of microbial populations to adapt to specific environmental conditions that influence the characteristics of the cheese. The study of microbial populations using culture-independent techniques allowed a better understanding of the bacterial community and differentiation in PDO cheeses. Further research is needed to better understand the diversity of these communities and their potential impact on human health.

## Author contributions

CS wrote the manuscript with support from SR and BB. All authors read and approved the final manuscript.

